# Comment on “Effect of Multiple Pharmacological Interventions and Magnetic Control on Capsule Endoscopy Gastrointestinal Transit Time and Diagnostic Yield: A Systematic Review and Meta‐Analysis”

**DOI:** 10.1002/jgh3.70361

**Published:** 2026-09-24

**Authors:** Nirupma Gupta, Sushma Narsing Katkuri, Varshini Vadhithala, Sachin Kumar, Sushma Verma, Jeffrin Reneus Paul

**Affiliations:** ^1^ Department of Anatomy, School of Medical Sciences and Research Sharda University Greater Noida India; ^2^ Department of Community Medicine Malla Reddy Instiute of Medical Sciences, Malla Reddy Vishwavidyapeeth, Suraram Hyderabad Telangana India; ^3^ Dr. D. Y. Patil Medical College, Hospital and Research Centre, Dr. D. Y. Patil Vidyapeeth (Deemed‐to‐be‐University) Pune Maharashtra India; ^4^ Faculty of Pharmaceutical Sciences Graphic Era Hill University Dehradun India; ^5^ Centre for Promotion of Research Graphic Era Deemed University Dehradun India; ^6^ Department of Pharmaceutics Noida Institute of Engineering & Technology (Pharmacy Institute) Greater Noida India; ^7^ Saveetha Medical College and Hospital, Saveetha Institute of Medical and Technical Sciences Saveetha University Chennai India

Dear Editor,

We carefully reviewed the systematic review and meta‐analysis by Kumar et al., which examined the influence of pharmacological agents and magnetic guidance on gastric transit time (GTT), small bowel transit time (SBTT), and completion rate (CR) in capsule endoscopy (CE). By synthesizing evidence from 53 studies involving over 9000 participants, the authors present one of the most comprehensive evaluations to date addressing strategies aimed at optimizing CE performance. This work addresses a clinically relevant and technically demanding aspect of small bowel assessment and therefore represents a valuable contribution to the field [1].

Notably, the analysis underscores the variable effects of agents such as metoclopramide, prucalopride, polyethylene glycol, castor oil, and magnetic steering systems, reinforcing the need for individualized rather than standardized preparation protocols. The observation that no single intervention uniformly improves all transit parameters is particularly relevant for clinical practice, where patient characteristics frequently influence procedural outcomes.

Nevertheless, several methodological issues merit further consideration. First, although multiple intervention categories with differing mechanisms of action were included, the analysis did not apply comparative synthesis approaches across these strategies. Adoption of a network meta‐analytic framework could have facilitated indirect comparisons and enabled clearer differentiation of the relative effectiveness among interventions [2].

Second, substantial statistical heterogeneity was observed across several pooled outcomes, particularly for GTT and SBTT, with *I* ^2^ values exceeding 90%. While the application of random‐effects models was appropriate, additional investigation using meta‐regression or stratified analyses based on clinical variables—such as diabetic status, prior abdominal surgery, hospitalization, or advanced age—might have provided further insight into the sources of variability across studies [3].

Third, assessment of publication bias relied primarily on visual inspection of funnel plots. Given that many outcomes were expressed as proportions, incorporation of alternative quantitative approaches, including Doi plots and the Luis Furuya–Kanamori (LFK) index, may have offered a more robust evaluation of potential small‐study effects [4].

Finally, although methodological quality was appraised using RoB 2.0 and the Newcastle–Ottawa Scale, the absence of a structured GRADE‐based assessment limits confidence in translating statistically significant findings into strong clinical recommendations, particularly in the context of marked heterogeneity across included studies [5].

Despite these considerations, the review provides important insights into CE optimization and supports the growing emphasis on patient‐tailored preparation strategies. Future investigations incorporating head‐to‐head randomized comparisons and advanced comparative meta‐analytic techniques will be instrumental in refining the selection of pharmacological and mechanical interventions for CE.

## Funding

The authors have nothing to report.

## Ethics Statement

The authors have nothing to report.

## Consent

The authors have nothing to report.

## Conflicts of Interest

The authors declare no conflicts of interest.

## Data Availability

Data sharing is not applicable to this article as no datasets were generated or analyzed during the current study.
